# Methotrexate and Adalimumab Decrease the Serum Levels of Cardiovascular Disease Biomarkers (VCAM-1 and E-Selectin) in Plaque Psoriasis

**DOI:** 10.3390/medicina56090473

**Published:** 2020-09-15

**Authors:** Natalia Zdanowska, Agnieszka Owczarczyk-Saczonek, Joanna Czerwińska, Jacek J. Nowakowski, Anna Kozera-Żywczyk, Witold Owczarek, Wojciech Zdanowski, Waldemar Placek

**Affiliations:** 1 Department of Dermatology, Sexually Transmitted Diseases and Clinical Immunology, The University of Warmia and Mazury, 10-229 Olsztyn, Poland; aganek@wp.pl (A.O.-S.); joannaj061@gmail.com (J.C.); w.placek@wp.pl (W.P.); 2 Department of Ecology and Environmental Protection, The University of Warmia and Mazury, 10-727 Olsztyn, Poland; jacek.nowakowski@uwm.edu.pl; 3 Department of Dermatology, Military Institute of the Health Services, 04-141 Warsaw, Poland; annakozera@o2.pl (A.K.-Ż.); witold.owczarek@dermedicus.pl (W.O.); 4 Department of Gynecology and Obstetrics, The University of Warmia and Mazury, 10-561 Olsztyn, Poland; wojciechzdanowskiw@gmail.com

**Keywords:** VCAM-1, E-selectin, psoriasis, methotrexate, adalimumab

## Abstract

*Background and objectives:* The shared pathogenesis of psoriasis and atherosclerosis may be determined by assaying the levels of endothelial activation molecules. This study aimed at evaluating vascular cell adhesion molecule 1 (VCAM-1) and E-selectin serum concentrations, and atherosclerosis severity in patients with plaque psoriasis. It also aimed to determine the effects of methotrexate/adalimumab treatment for 12 weeks on the plasma levels of the aforementioned molecules. *Materials and Methods:* The study included 34 psoriasis patients (17 treated with methotrexate and 17 treated with adalimumab) and eight controls. The 10-year risk of a fatal cardiovascular disease, body mass index, Psoriasis Area and Severity Index, and body surface area were calculated for each subject. VCAM-1 and E-selectin levels were determined via an enzyme-linked immunosorbent assay at baseline and after 12 weeks. *Results:* Baseline E-selectin and VCAM-1 levels were higher in the adalimumab group than in the methotrexate and control groups. VCAM-1 levels decreased in the adalimumab (*p* = 0.02) and methotrexate groups (*p* = 0.008), while E-selectin levels decreased in the methotrexate group (*p* = 0.004). *Conclusions:* The results indicate a correlation between systemic psoriasis treatment and E-selectin and VCAM-1 plasma concentrations, which may be associated with the risk of cardiovascular disease development.

## 1. Introduction

Epidemiological data have provided evidence of an association between psoriasis and adverse cardiovascular outcomes [1]. This association may be determined by assessing their shared pathogenesis involving endothelial dysfunction [2,3]. Vascular cell adhesion molecule 1 (VCAM-1) is an inducible glycoprotein, and E-selectin is a soluble cell adhesion molecule. Both molecules are primarily expressed in the endothelium [4,5,6,7]. The serum levels of soluble VCAM-1 appear to be correlated with the degree of atherosclerosis and may be used for diagnosing the early stages of the condition [8]. Endothelial activation, resulting in E-selectin expansion, induces leukocyte rolling along the vascular wall and mediates inflammation in various diseases, including atherosclerosis [9]. A common feature of the inflammatory process in psoriasis and atherosclerosis is leukocyte migration, to which VCAM-1 and E-selectin significantly contribute. These adhesion molecules, being the indicators of endothelial activation, might be potential biomarkers of inflammatory activity and of the severity of atherosclerosis and cardiovascular disease coexisting with psoriasis [10].

In clinical practice, atherosclerosis and its severity are assessed by determining the 10-year risk of fatal cardiovascular disease, which is estimated using the European Risk Chart: Systematic Coronary Risk Evaluation (SCORE) [11,12]. Methotrexate (MTX) is used to treat psoriasis, and one of its main mechanisms of action is believed to be based on its effect of decreasing E-selectin expression [13,14]. Endothelial function, which is significantly altered in psoriasis patients, may also improve during treatment with tumor necrosis factor alpha (TNF-alpha) inhibitors [15].

This study aimed at determining VCAM-1 and E-selectin levels and evaluating their relationship with psoriasis severity compared to those in healthy controls. We also estimated atherosclerosis severity by evaluating cardiovascular risk in patients with plaque psoriasis who were assigned to receive a systemic treatment. The objective was also to assess the impact of 12-week MTX and adalimumab (ADA) treatments on the VCAM-1 and E-selectin concentrations in psoriasis patients. We believe our research will supplement information concerning cardiovascular and atherosclerosis risks in patients with plaque psoriasis, as well as the effects of MTX and ADA on endothelial activation markers in psoriasis. Understanding the impact of systemic psoriasis treatment on E-selectin and VCAM-1 serum levels will enable the selection of the appropriate therapy for both cardiovascular disease and skin lesions as coexisting conditions.

## 2. Materials and Methods

### Study Group

This prospective cohort study was conducted in 34 patients (27 men and seven women) with plaque psoriasis (age: 30–73 years) and eight healthy volunteers (age: 30–57 years) as the control group. The study was approved by the Bioethics Commission at the University of Warmia and Mazury in Olsztyn, Poland (Resolution 16/2019). Each patient included in the study provided informed consent for participation. The inclusion criteria included being of an age higher than 18 years and a diagnosis of moderate-to-severe plaque psoriasis. The exclusion criteria were as follows: age below 18 years, mild plaque psoriasis, pregnancy, breastfeeding, and previous biological treatment (in the case of patients who qualified for MTX treatment). Due to the requirements of the treatment program for moderate-to-severe plaque psoriasis in Poland, patients starting ADA treatment confirmed the contraindications and/or side effects associated with the use of at least two classic methods of systemic psoriasis treatment or a history of ineffective systemic treatment. The systemic treatment is understood as the use of at least two of the following agents: MTX, cyclosporine, acitretin, and psoralen ultraviolet A (PUVA).

Of the patients, 17 were treated only with MTX and the remaining 17 were treated with ADA only. The patients received oral MTX at a dose of 7.5–20 mg per week and subcutaneous ADA at an initial dose of 80 mg followed by 40 mg every 2 weeks. The observation period lasted 12 weeks. The following parameters were evaluated for each subject: body mass index (BMI), the severity of psoriasis based on the Psoriasis Area and Severity Index (PASI), and body surface area (BSA). Depending on risk factors, such as age, sex, systolic blood pressure, smoking, and total cholesterol, the risk of fatal cardiovascular disease events over the next 10-year period was estimated using SCORE charts [16]. Laboratory tests to determine E-selectin and VCAM-1 serum levels were performed via an enzyme-linked immunosorbent assay (commercial ELISA kit, EIAab Science Co., Ltd., Wuhan, China) by using fasting blood samples obtained before and after 12 weeks of treatment. 

Statistical analyses were performed using Statistica 13.1 (StatSoft Poland). Mean values and standard deviations (±SD) were used to describe the level of variables (PASI, BSA, VCAM-1, E-selectin) and demographic characteristics of the studied groups. The distributions of all studied explanatory metric variables in the groups were compared to the normal distribution using the Shapiro–Wilk test, and the homogeneity of variance was tested using the Bartlett test. The one-way ANOVA model was used when the important assumptions for the analysis of variance were met. In case of failure to meet the assumption with a distribution close to normal, the Kruskal–Wallis test was used. The Mann–Whitney test was used to test the differentiation of variable levels between two groups: MTX vs. control and ADA vs. control. Comparisons of variables over time (baseline (W0) vs. the end of the study (W12)) were made using the paired samples Student’s T-test if a parametric analysis was possible. For other cases, the nonparametric Wilcoxon test was used. Correlations were analyzed using Spearman’s correlation coefficient. The χ^2^ test was used to compare the distribution of two demographic nominal characteristics (sex, smoking) between the experimental and control groups. The Kruskal–Wallis test was also used for the comparison of the level of VCAM-1 and E-selectin between four groups of patients classified as presenting different SCORE risk levels. Differences and correlations were considered statistically significant at *p* < 0.05. 

## 3. Results

The demographic data of the patients included in the study are listed in Table 1. BMI was significantly higher in the psoriasis group than in the control group (*p* = 0.001). Likewise, the BMI in psoriasis patients was significantly and positively correlated with PASI and BSA (Spearman’s correlation coefficients 0.5 and 0.56; *p* = 0.0007 and *p* = 0.0001, respectively). The healthy volunteers were nonsmokers. They were significantly younger (*p* = 0.01), and had significantly lower systolic blood pressure (*p* = 0.04) than the patients qualifying for MTX treatment. Therefore, the 10-year risk of fatal cardiovascular disease and atherosclerosis risk (estimated via the European Risk Chart SCORE) among the healthy volunteers were significantly lower than the corresponding risks among psoriasis patients assigned to receive MTX (*p* = 0.001). 

**Figure 1:**
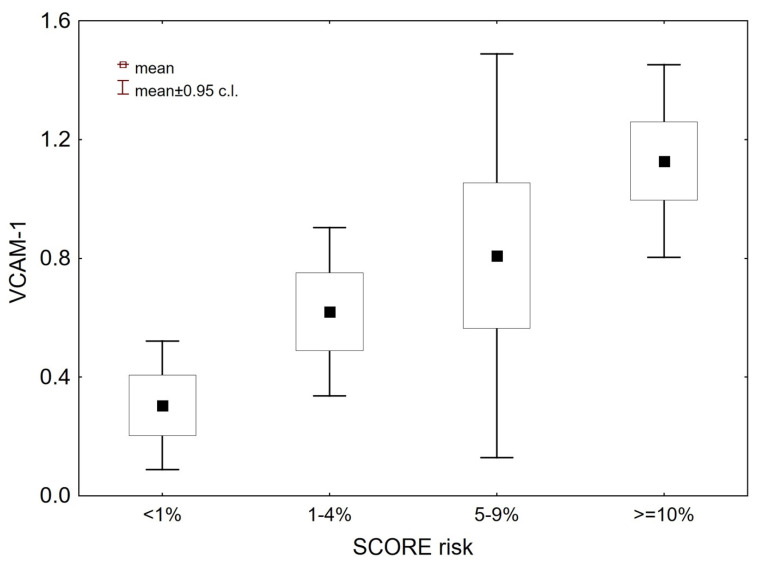
Vascular cell adhesion molecule 1 serum levels in patients with ten-year risk of fatal cardiovascular disease estimated via SCORE charts. Abbreviations—VCAM-1: vascular cell adhesion molecule 1, SCORE risk: ten-year risk of fatal cardiovascular disease estimated via SCORE (Systemic Coronary Risk Evaluation) charts.

**Figure 2:**
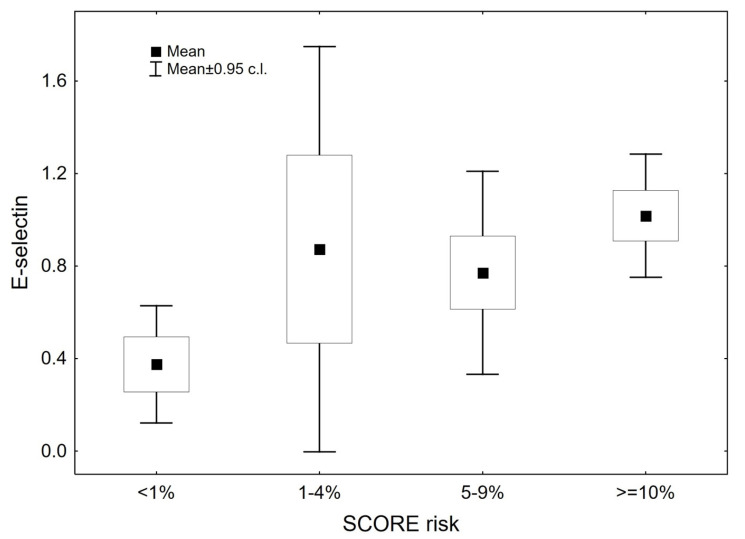
E-selectin serum levels in patients with ten-year risk of fatal cardiovascular disease estimated via SCORE charts. Abbreviations—SCORE risk: ten-year risk of fatal cardiovascular disease estimated via SCORE (Systemic Coronary Risk Evaluation) charts.

Figure 1 and Figure 2 show the levels of VCAM-1 and E-selectin in patients depending on the ten-year risk of fatal cardiovascular disease estimated via SCORE charts. At baseline, the serum levels of VCAM-1 and E-selectin were significantly higher in patients with an estimated SCORE risk >= 10% compared to patients with a risk of <1% (*p* = 0.02 and *p* = 0.012, respectively).

The evaluation of the severity of psoriasis is shown in Table 2. Patients who qualified for treatment with ADA presented more severe psoriatic lesions than those who qualified for MTX therapy. As shown in Table 3, baseline VCAM-1 and E-selectin levels were significantly correlated with disease activity (PASI and BSA) in psoriasis patients. Interestingly, when examining only the patients assigned for treatment with MTX or ADA, the correlation between these values was not significant. Table 4 shows the VCAM-1 and E-selectin levels at W0 and W12 in groups of patients treated with MTX and ADA. In the control group, only W0 values were noted, as the group included healthy volunteers who did not require treatment.

At W0, the highest levels of VCAM-1 and E-selectin were found in the subjects assigned to receive ADA, and the lowest levels were noted in the control group. Regarding the plasma levels of VCAM-1, a comparison between the groups showed significant differences among patients starting treatment with ADA versus MTX (*p* = 0.01) and versus the control group (*p* = 0.000002). Differences were also significant between patients starting treatment with MTX and the control group (*p* = 0.02). With regard to the levels of E-selectin, significant differences were found among patients starting treatment with ADA versus MTX (*p* = 0.027) and versus the control group (*p* = 0.00001). Furthermore, significant differences were observed in patients starting treatment with MTX versus the control group (*p* = 0.034). At W12, the difference between the MTX and ADA groups in terms of the levels of both E-selectin (*p* = 0.000005) and VCAM-1 (*p* = 0.000005) was also significant. A significant decrease was noted in the VCAM-1 serum levels (W0 vs. W12) in patients treated with ADA (*p* = 0.02) or MTX (*p* = 0.008). The reduction in E-selectin level was significant only in patients treated with MTX (*p* = 0.004).

## 4. Discussion

The results of our study highlight a relationship between the severity of psoriasis as described via PASI and BSA with BMI and the levels of VCAM-1 and E-selectin. They also prove the relationship between the level of studied particles and the ten-year risk of fatal cardiovascular events estimated via SCORE charts. A significant decrease in the serum levels of both studied adhesion molecules was observed in patients receiving MTX. However, only VCAM-1 levels decreased in patients treated with ADA.

The interaction of endothelial adhesion molecules (such as VCAM-1) with selectins (including E-selectin) mediates the migration of activated T cells and macrophages from blood vessels. It also initiates adherence between the vascular endothelium and neutrophils, monocytes, eosinophils, and T lymphocytes during the inflammatory process, resulting in plaque formation in both psoriasis and atherosclerosis [17,18,19]. Several studies showed higher levels of E-selectin and VCAM-1 in psoriasis [19,20,21,22]. The E-selectin serum level is correlated with the severity of psoriasis (as indicated by PASI), so it appears to be an indicator of disease activity [21]. Furthermore, Szepietowski et al. confirmed that the plasma levels of E-selectin significantly decreased after treatment in psoriasis patients [19].

The present study demonstrated the highest VCAM-1 and E-selectin levels in the group of patients assigned to receive ADA, lower values in patients beginning MTX treatment, and the lowest values in the control group. Baseline VCAM-1 and E-selectin levels were significantly and negatively correlated with PASI and BSA in psoriasis patients. Interestingly, the correlation between these levels was not significant after the patients were divided into MTX and ADA treatment groups. Similarly, BMI was significantly correlated with the severity and extent of skin lesions (as indicated by PASI and BSA) in psoriasis patients. No correlation of BMI with either VCAM-1 or E-selectin was found. A high BMI appears to be positively correlated with the severity of psoriasis and defines the clinical response to systemic treatment [23,24,25]. Long-term anti-TNF-alpha therapy was related to BMI elevation in psoriasis patients [26,27].

Few reports demonstrated the correlation of adhesion molecules with BMI. Most of them concerned E-selectin, whose level positively correlated with BMI [28,29,30]. However, Abd El-Kader et al. demonstrated that a reduction in BMI resulted in the modulation of VCAM-1 levels in obese patients with type 2 diabetes [31]. The essential mechanism of action of MTX in psoriasis might relate to a decrease in E-selectin expression during treatment. Torres-Alvares et al. showed that a reduced expression of E-selectin and VCAM-1 in the blood vessels of psoriasis patients after therapy [13,14]. Endothelial function, which is significantly altered in psoriasis patients, may also improve during treatment with TNF-alpha inhibitors. Furthermore, several studies provided evidence of a decrease in E-selectin levels in the sera of psoriasis patients during treatment with infliximab or ADA [2,10,15,32].

In our study, E-selectin and VCAM-1 levels significantly decreased during MTX treatment. Moreover, contrary to E-selectin levels, the decrease in VCAM-1 was significant in patients treated with ADA. The results suggest that endothelial function and the cardiovascular risk of patients with plaque psoriasis are influenced to a larger extent by MTX than ADA.

The most significant limitations of our study were the low number of patients (34 psoriasis patients and eight healthy volunteers), short observation time (12 weeks), and volunteer self-selection of the control group. Volunteer serum samples were not obtained for VCAM-1 and E-selectin determination at W12 because, as healthy individuals, they were not treated. The present results will be verified in a larger study group over an extended period to draw more general conclusions, including the impact of systemic therapy of psoriasis on cardiovascular risk.

## 5. Conclusions

The results of our study demonstrate a possible impact of psoriasis itself and its systemic treatment on serum E-selectin and VCAM-1 levels. Therefore, they indicate the risk of developing cardiovascular disease. VCAM-1 and E-selectin serum levels appear to be correlated with psoriasis severity as indicated by PASI and BSA. After 12 weeks of MTX administration, E-selectin and VCAM-1 levels significantly decreased. However, treatment with ADA resulted in a significant decrease only in VCAM-1 levels. Therefore, compared to ADA, MTX may have a greater impact on the levels of adhesion molecules, so it might help determine the risk of cardiovascular disease development in psoriasis patients.

## Figures and Tables

**Table 1:** Demographic characteristics of the study groups at baseline (W0).

	MTX (*n* = 17)	ADA (*n* = 17)	Control (*n* = 8)	*p*
Age (years) (mean ± SD)	33–73 (52.9 ± 11.97)	24–72 (46.1 ± 13.55)	30–57 (34.6 ± 9.38)	0.005 ^1^
				0.25 *
				0.01 **
				0.15 ***
Sex (M/F)	14/3	13/4	2/6	0.01 ^2,4^
Height (cm) (mean ± SD)	158–190 (174.2 ± 8.49)	161–186 (174.6 ± 20.62)	160–192 (172.4 ± 11.59)	0.832 ^3,6^
Weight (kg) (mean ± SD)	63–129 (88.3 ± 17.29)	60–115 (93.4 ± 25.40)	48–98 (68.6 ± 19.03)	0.014 ^3^
				0.58 *
				0.18 **
				0.01 ***
BMI (kg/m^2^) (mean ± SD)	23.1–39.8 (29 ± 5.24)	24–42.4 (30.7 ± 8.69)	19.1–33 (22.6 ± 5.14)	0.001 ^1^
				0.57 *
				0.03 **
				0.005 ***
Cholesterol, total (mg/dL) (mean ± SD)	144–303 (212 ± 44.52)	144–276 (203 ± 43.28)	173–244 (210.5 ± 24.63)	0.805 ^1,5^
Smoking: yes/no	10/7	5/12	0/8	0.01 ^2,4^
Systolic blood pressure (mmHg) (mean ± SD)	125–177 (140.9 ± 16.4)	109–156 (134.4 ± 13.35)	110–135 (123.6 ± 9.4)	0.049 ^3^
				1.0 *
				0.04 **
				0.2 ***
SCORE (%) (mean ± SD)	<1–10 (6.9 ± 7.36)	<1–8 (3.2 ± 2.23)	<1–1 (1 ± 0)	0.0012 ^3^
				0.31 *
				0.001 **
				0.07 ***

Abbreviations: MTX: patients qualified for treatment with methotrexate; ADA: patients qualified for treatment with adalimumab; BMI: body mass index; SCORE: estimation of the risk of fatal cardiovascular disease events over the next 10-year period via Systematic Coronary Risk Evaluation charts. ^1^ One-way ANOVA model, ^2^ χ^2^ test, ^3^ Kruskal–Wallis tests, ^4^ nonrandomized group structure, ^5^ no level of differentiation between the study groups, ^6^ no significant differentiation was found by testing the hypothesis with the Kruskal–Wallis test, and there are no post hoc results, * post hoc test: MTX vs. ADA, ** post hoc test: MTX vs. control, *** post hoc test: ADA vs. control.

**Table 2:** Severity of psoriasis (Psoriasis Area and Severity Index (PASI) and Body Surface Area (BSA)) during the study period.

	MTX (*n* = 17)		ADA (*n* = 17)	
	W0	W12	W0	W12
PASI (mean ± SD)	6.5–26.2 (12 ± 5.0)	0–15 (4.5 ± 4.05)	12.1–33.2 (20.6 ± 5.41)	0–11.8 (3.6 ± 3.11)
	*p* = 0.001 ^1^		*p* = 0.0003 ^1^	
BSA (%) (mean ± SD)	10–46 (19.8 ± 9.43)	0–20 (6.9 ± 6.30)	12.5–77 (33.8 ± 17.57)	0–22.5 (8.9 ± 8.77)
	*p* = 0.002 ^1^		*p* = 0.0005 ^1^	

Abbreviations: PASI: Psoriasis Area and Severity Index; BSA: body surface area; W0: baseline; W12: end of the study; ^1^ Wilcoxon test.

**Table 3:** Correlation of VCAM-1 and E-selectin levels with PASI and BSA at W0.

Values at Baseline (W0)		Ps (*n* = 34)		MTX (*n* = 17)		ADA (*n* = 17)	
		Spearman’s Correlation Coefficient	*p*	Spearman’s Correlation Coefficient	*p*	Spearman’s Correlation Coefficient	*p*
VCAM-1	PASI	−0.4	0.01	−0.21	0.4	−0.07	0.76
	BSA	−0.38	0.02	−0.15	0.54	−0.21	0.4
E-selectin	PASI	−0.4	0.01	−0.27	0.29	−0.06	0.81
	BSA	−0.35	0.03	−0.21	0.4	−0.1	0.7

Abbreviations: VCAM-1: vascular cell adhesion molecule 1; Ps: psoriasis patients; MTX: patients qualified for treatment with methotrexate; ADA: patients qualified for treatment with adalimumab; PASI: Psoriasis Area and Severity Index; BSA: body surface area.

**Table 4:** VCAM-1 and E-selectin serum levels at W0 and W12.

		Methotrexate		Adalimumab		Control	Comparisons between the Groups		
		Mean (ng/mL) ± SD	*p* (W0 vs. W12)	Mean (ng/mL) ± SD	*p* (W0 vs. W12)	Mean (ng/mL) ± SD	MTX vs. ADA (*p*)	MTX vs. C (*p*)	ADA vs. C (*p*)
VCAM-1	W0	0.45 ± 0.32	0.008 ^1^	1.03 ± 0.46	0.02 ^1^	0.04 ± 0.03	0.01 ^2^	0.02 ^2^	0.000002 ^2^
	W12	0.2 ± 0.22		0.91 ± 0.33		N/A	0.000005 ^3^	N/A	N/A
E-selectin	W0	0.46 ± 0.39	0.004 ^1^	1.24 ± 1.26	0.16 ^1^	0.04 ± 0.05	0.027 ^2^	0.034 ^2^	0.00001 ^2^
	W12	0.16 ± 0.19		0.9 ± 0.33		N/A	0.000005 ^3^	N/A	N/A

Abbreviations: VCAM-1: vascular cell adhesion molecule 1; W0: baseline; W12: end of the study; MTX: patients qualified for treatment with methotrexate; ADA: patients qualified for treatment with adalimumab; C: control group. ^1^ Wilcoxon test, ^2^ Kruskal–Wallis test, and ^3^ Mann–Whitney test.
